# The Institutional Library

**Published:** 1918-10-05

**Authors:** 


### THE INSTITUTIONAL LIBRARY—(*continued*).

### National Reconstruction: A Study in Practical Politics and Statesmanship.*

THIS is a book for those who think, who have ideas and do not dislike mental labour to find fresh ones. The book teems with ideas, but the obscurity of the style makes it difficult to get at the thoughts. Long, involved sentences full of metaphor and allusion and an indefiniteness as to which noun a pronoun refers make it a trying book to read. The closest attention is necessary to extract the great value that is in it. There is much oracular. There is a plentiful sowing of pithy, illuminating sentences. There is not a definite scheme of reconstruction to be found in the book, and no intention to formulate one.

Mr. Robinson understands the vital necessity of definition of terms, but does he succeed in defining? National reconstruction is the effective application of progressive knowledge to national development. National development is that which stimulates and fixes integrating growth in the units which make up a nation; and the terms are further defined in the first pages in the book. He warns us that things are as they are, and it follows that what is must be the tools and the materials with which the reconstructive work must be done. He hopes that the constructive work can be done by adjustable will rather than through revolutionary rendings. This is a matter on which living nations have their choice. To judge by what we see now in Russia, the attempt at reconstruction through revolutionary rendings vastly increases the amount of reconstruction to be done. It is the object of this book to adumbrate the manner in which things as they are may be led on towards the goal of things as they should be. Exactly how is not set forth, but the instruments are "national constructors." The English race has been stated to be forty or fifty million people, mostly fools. This view extended to all mankind seems to be that held by the author; probably he is correct in his view. He seems to aim at the wise minority standing in the crowd and a little apart from it, feeling its superiority to the ruck, slightly contemptuous and full of pity for the blind groping with good intent round it, with good nature and amusement gently directing the blind groping and carefully edging the crowd the way it should go. He sees for the minority much interest and amusement in this philanthropic direction, and even material profit, but certainly satisfaction. They shall be as gods in Olympus watching, directing, and diverted. If great men, all may be well; but what if they be but superior persons? There is danger of that.

An important part of the machinery to be used is to be questions. Clear, definite statements of objects are to be asked of every legislator or legislative party that makes any proposal. Certainly no more embarrassing way of dealing with a politician can be conceived than to ask him, and to insist on being clearly answered, What is your exact object? and to supplement that question by others of searching, probing quality.

The first piece of reconstruction should be of the fighting forces, on the grounds that an Empire that is not secure can neither be constructed nor developed. That is soundest sense. A nation not confident it can keep itself safe from possible aggression must be always looking over its shoulder. That interferes with work. The author seems to recognise that Navy and Army training is good discipline for the individual, enlightening and educating mentally, and making for physical health and efficiency. He pours scorn on those who refuse acceptance of national duty and desire a comfortable earth for themselves as a prelude to a still more comfortable and unmerited continuation hereafter. There is so much pith in the book that it is not possible to do more than indicate what may be found in it. It is well worth the toil of attentive reading.

* By J. J. ROBINSON. (London: Hurst and Blackett, Ltd. Price 2s. 6d. net.)

**Glaucoma.** A Text-Book for the Student of Ophthalmology. By LT.-COL. R. H. ELLIOT, M.D. Pp. xvi. + 546. 158 Illustrations. (London: H. K. Lewis. Price 21s. net.)

THIS volume consists mainly of an exhaustive *résumé* of the facts and theories bearing on the subject of glaucoma. As such it should take a place in the library of every student of ophthalmology, who will find in it a vast deal of information hitherto obtainable only by reference to many books and pamphlets. The author emphasises the points which he considers to be of most importance, and, where differences of opinion exist, indicates that to which he inclines, giving his reasons for so doing. The subject is treated from every point of view, anatomical, clinical, and therapeutic, these subdivisions being indicated by the headings of the various chapters. Authors and research-workers are freely quoted, and valuable assistance for further reading is given by a full bibliography for each section, as well as by an index of names at the end of the book. The diagnosis of glaucoma is specially fully discussed, and a useful section is that which deals with the changes which take place in the visual fields. The book concludes with a history and description of the various operative measures which have been devised for the relief of the condition, the author's own operation being minutely described in its successive steps. The illustrations are numerous and helpful.

**War Wounds of the Lung.** By PIERRE DUVAL. With 27 Plates and Illustrations. Authorised English Translation. (Bristol: John Wright and Sons. 1918. Pp. 99 + viii. Price 8s. 6d. net.)

GOOD books are rare, good small ones rarer, good medical small ones—or so a reviewer tends to think—rarest of all. Yet this is a good one, although it must, of course, in time be superseded. The feature of the work is the thoroughgoing application of ordinary surgical principles to severe wounds of the lung by shell fragments, with a success which suggests a further extension of surgery to medical pulmonary disease. However, the patients of Dr. Duval and his colleagues were of course healthy young men, although men weakened by severe trauma. All things considered, the absence of shock when, for instance, large pleural adhesions were rapidly broken down is remarkable: previous experience in lung collapse therapy of consumption had pointed in the other direction. Indeed, the modern phthisio-therapeutist will find much of interest in these very practical pages, which are illustrated to advantage. The translation is well done. Necessarily a field manual, it takes little account of after-history and sequelæ; or of bibliography—as, for instance, the paper of Philipowicz in a Vienna medical journal last year. It impresses the reader, however, as a likely factor in the formation of authoritative opinion on its subject.

**Institutional Library**—(*continued*).

**First Aid in Emergencies.** By Eldridge L. Eliason, M.D. (Philadelphia and London: J. B. Lippincott Co. Second Edition, enlarged. Price 6s. net.)

The first thing which strikes us on handling this book is the facility with which any particular subject may be run to earth by the person faced with a sudden emergency. There is a comprehensive synopsis of the various chapters, a capital detailed index, a thumb-nail index, and a paged list of illustrations which number 106. This is as it should be. The contents of a book on Emergencies, Medical and Surgical, should be, like those of an emergency bag, easily accessible. The number of volumes on this subject must be legion, but the fact that a second edition of the one under review has been called for would seem to indicate it is not a work of supererogation. It is somewhat more comprehensive in scope than most of its kind, and whilst the larger boundary allowed himself by the author takes in ground not necessary, or even advisable, for the sheer amateur to tread, the book may have found a public amongst those who wish to obtain a really sound grasp of the principles involved in any given catastrophe. For such the work should prove valuable, provided always they bear in mind the injunction, "Send for a doctor," by which the author safeguards himself in dealing with treatment. From a literary point of view the less said the better, but how often this obtains in the writings of medical men! There are numerous uncouth expressions; the style generally is slapdash and untidy; and carelessness in punctuation ofttimes renders absurd (occasionally diverting) a sentence which otherwise would be at least intelligible.

**Notes on Venereal Diseases for Nurses and Midwives.** By Dr. Mary Scharlieb. (London: The Scientific Press, Ltd. 1918. Pp. 135 + vi. Price 1s. 3d. net.)

Recent developments in the social and professional recognition of the serious national consequences of venereal diseases make it inevitable that nurses should receive some additional instruction in the subject. They have their part to play in the organised treatment centres, and those who act as midwives may well have special opportunities of observing cases in an early and curable stage. To afford the required instruction is not an easy task. The audience has not the preliminary training necessary for the understanding of technicalities, and, further, there is the danger that teaching which is intended for guidance in a subordinate *rôle* may be regarded by the pupils as the justification for a main responsibility. These difficulties are really substantial, and for the most part Mrs. Scharlieb has met them with excellent judgment and much literary skill. Certainly she has produced a brief but lucid and effective sketch of the principal clinical features of syphilis and gonorrhœa, and nurses who may be brought into contact with cases of this order may read her book with great advantage. In one or two places the note is not quite clear that nurses have only one function to discharge in reference to treatment—namely, the application of the orders given by a medical practitioner—and there is some risk of misconstruction in the remark that, because patients and friends consider them to be authorities on medical subjects nurses should "equip themselves with the knowledge that alone can make their advice really valuable." The only "valuable advice" in such circumstances is to direct the patient to consult a doctor, and a nurse who knows her duty, whether she has little knowledge or much, will give no other. Considering the temptations to which nurses are subjected in this relation, anyone who aspires to guide them cannot be too emphatic on the point. Mrs. Scharlieb, we are sure, appreciates the danger here suggested, and if we had any doubt on the point we should find it resolved by the fact that in her preface she is not content with Pope's well-known line, "A little learning is a dangerous thing," but for "learning" substitutes "knowledge." With a mind so alert to the risks of encouraging her readers to tasks for which they are quite unfitted, we think her definition of the dangers somewhat wanting in precision and emphasis. If we may venture one other critical word, it would be to deplore the split infinitives. Yet we would not be thought wanting in appreciation of a difficult and delicate task, accomplished, we are sure, after much careful consideration and with a most sincere motive. Mrs. Scharlieb will have many readers, and her audience will be a grateful one.

**Thyrea and Other Sonnets.** By John Ferguson. (London: Andrew Melrose. 1918. Price 1s. net.)

The sonnet sequence with which this slender volume opens was written in a sanatorium. One of the series, Number IV., if we remember, has already appeared in The Hospital. Since it is easier to attempt a short poem than a long one, the sonnet is rightly popular with minor poets. There are few supremely good sonnets, but many passable or excellent, and of the latter class we find several here. Hospital life has inspired the Muse too rarely, and we are grateful for such lines as the following, which give a direct and sincere picture of certain aspects of a patient's life in the ward.

"They do not like us to die here, we know," Mr. Ferguson slily makes one character remark, and we seem to recognise that fellow-patient who

"Was by his ominous cough endeared and known."

He describes his fellow-patients in "L'Envoi," perhaps the best of the series, as:

"So old in Sorrow they, and yet so young,
My hopeless brothers lying prostrate here."

There is eloquence, too, in the first half of "Stella Maris," but the writer is less successful in his impressionistic sketches, where the slang and the colloquialisms do not achieve the vividness at which they aim, and, like other vulgarities, have no intrinsic quality to recommend them. He should be grateful for Mr. W. L. Courtney's discreet preface, and to the encouragement given to him there we would add our own belief that if the writer will be content with nothing but his own best, the hospital world may come to remember him in the train of Whitman and Henley, in spite of whom the hospital has hardly yet found its place on the map of English literature.

